# Reducing tumor growth and angiogenesis using a triple therapy measured with Contrast-enhanced ultrasound (CEUS)

**DOI:** 10.1186/s12885-015-1333-7

**Published:** 2015-05-08

**Authors:** Philipp Marius Paprottka, Svenja Roßpunt, Michael Ingrisch, Clemens C Cyran, Konstantin Nikolaou, Maximilian F Reiser, Brigitte Mack, Olivier Gires, Dirk A Clevert, Pamela Zengel

**Affiliations:** 1 Institute for Clinical Radiology, Ludwig Maximilian University Hospital, Munich, Germany; 2 Institute for Ear, Nose and Throat Medicine, Ludwig Maximilian University Hospital, Munich, Germany

**Keywords:** Contrast-enhanced ultrasound (CEUS), Experimental squamous cell carcinoma, VueBox

## Abstract

**Background:**

To evaluate the in vivo response by detecting the anti-angiogenic and invasion-inhibiting effects of a triple-combination-therapy in an experimental-small-animal-squamous-cell-carcinoma-model using the “flash-replenishment” (FR) method to assess tissue hemodynamics via contrast-enhanced-ultrasound (CEUS).

**Methods:**

Human hypopharynx-carcinoma-cells were subcutaneously injected into the left flank of 22-female-athymic-nude-rats. After seven days of subcutaneous tumor growth, FR-measurements were performed on each rat. Treatment-group and control-group were treated every day for a period of one week, with the treatment-group receiving solvents containing a triple therapy of Upamostat®, Celecoxib® and Ilomastat® and the control-group solvents only. On day seven, follow-up measurements were performed using the same measurement protocol to assess the effects of the triple therapy. VueBox® was used to quantify the kinetic parameters and additional immunohistochemistry analyses were performed for comparison with and validation of the CEUS results against established methods (Proliferation/Ki-67, vascularization/CD31, apoptosis/caspase3).

**Results:**

Compared to the control-group, the treatment-group that received the triple-therapy resulted in a reduction of tumor growth by 48.6% in size. Likewise, the immunohistochemistry results showed significant decreases in tumor proliferation and vascularization in the treatment-group in comparison to the control-group of 26%(p≤0.05) and 32.2%(p≤0.05) respectively. Correspondingly, between the baseline and follow-up measurements, the therapy-group was associated with a significant(p ≤ 0.01) decrease in the relative-Blood-Volume(rBV) in both the whole tumor(wt) and hypervascular tumor(ht) areas (p≤0.01), while the control-group was associated with a significant (p≤0.01) increase of the rBV in the wt area and a non-significant increase (p≤0.16) in the ht area. The mean-transit-time (mTT) of the wt and the ht areas showed a significant increase (p≤0.01) in the follow-up measurements in the therapy group.

**Conclusion:**

The triple-therapy is feasible and effective in reducing both tumor growth and vascularization. In particular, compared with the placebo-group, the triple-therapy-group resulted in a reduction in tumor growth of 48.6% in size when assessed by CEUS and a significant reduction in the number of vessels in the tumor of 32% as assessed by immunohistochemistry. As the immunohistochemistry supports the CEUS findings, CEUS using the “flash replenishment”(FR) method appears to provide a useful assessment of the anti-angiogenic and invasion-inhibiting effects of a triple combination therapy.

## Background

Occult tumor cells that can lead to loco-regional recurrence and distant metastases are the main reason for the poor prognosis of patients suffering from squamous cell carcinoma of the head and neck [1]. Tumor cells utilize a complex set of molecular mechanisms in order to metastasize [2]. Invasive migration through and remodeling of surrounding tissue is achieved upon degradation of the extracellular matrix (ECM). In this respect, the urokinase-type plasminogen-activator system (uPA) and matrix metalloproteases (MMP) are of major importance. The activity of MMPs and the uPA fosters cell migration, angiogenesis and metastasis [3,4]. Tumors greater than 1.5 mm^3^ in size require intimate contact with blood vessels or otherwise become necrotic [5]. Neoangiogenesis, *i.e.* the formation of new blood vessels, is provided *via* the production of chemoattractans, which redirect endothelial cells into the tumor tissue to enable tumor progression. In turn, de novo formed vessels enhance tumor invasion and metastasis through the production of MMP 2 and 9 and uPA, which further degrade ECM. The in vivo anti-metastatic and anti-proliferative activity of the synthetic uPA inhibitor WX-UK1 has been demonstrated in various animal tumor models [6], especially the suppression of rat breast cancer metastasis and reduction of primary tumour growth by the small synthetic urokinase inhibitor WX-UK1 [7].

WX-UK1 is the active metabolite of the oral prodrug Upamostat® administered as a component of the triple therapy in this study. Inhibition of MMPs has provided a significant increase in the survival rate in clinical trials, according to the frequent overexpression of MMPs in malignant tumors and the correlation with a highly aggressive phenotype and poor prognosis [8-10]. Combinatorial inhibition of MMPs and the plasminogen activator system using siRNA approaches likewise revealed effectiveness with a 60% and 90% down-regulation of invasion and angiogenesis, respectively [11,12]. Non-steroidal anti-inflammatory drugs are a group of pharmaceutical agents with anti-angiogenic properties. In vitro studies suggested a potential of cyclooxigenase-2 (COX-2) inhibitors to reduce the growth of colon, head and neck, and skin tumors and to block angiogenesis [13,14].

Former studies using the serine protease inhibitor WX-UK1, in which the effects on the capacity of tumor cell spheroids to invaginate and invade fibroblast spheroids were tested, present a reduction of tumor cell invasion of 50% using WX-UK1 alone [15]. Previous experience disclosed that only multimodal therapy strategies properly take into account the plethora of mechanisms underlying tumor progression and are hence indispensable. Thus, a promising concept is a combination of inhibitors that address different aspects of tumor progression and metastasis formation [16,17].

A previous in vitro study, which combined the serine protease inhibitor WX-UK1, the MMP inhibitor Ilomastat® and the selective COX-2 inhibitor Celecoxib®, demonstrated an inhibition of tumor cell invasion in a spheroid model of 80% and inhibition of angiogenesis by 40% in a HUVEC sprouting model [18]. The degree of neoangiogenesis is crucial for tumor growth and the propensity for forming metastasis. A number of molecular drugs promise to be effective at inhibiting tumor angiogenesis.

Established methods of monitoring therapy, such as assessing the size and growth behavior of a tumor during therapy (using *RECIST criteria*) or progression-free survival of patients, are not sensitive enough and not sufficiently specific to detect the subtle effects of these new molecular therapeutics in the early stages of therapy. Multiple preclinical studies, with varying degrees of success, have attempted to display different functional parameters of tumor microcirculation and for therapy monitoring of an anti-angiogenic treatment [19-23], e.g. by means of contrast-enhanced ultrasound imaging.

One major advantage of CEUS is its non-invasive nature allowing for the depiction of various organs with high spatial and temporal resolution without the use of ionizing radiation. Ultrasound contrast agents (e.g. Sonovue®) contain a gas that is exhaled via the lungs such that elimination from the body is usually ensured within a few minutes. In contrast to iodine and gadolinium, these ultrasound contrast agents are not eliminated via renal excretion, so they are not contra-indicated for patients with impaired renal function.

Conventional, indicator-based methods for the assessment of tissue hemodynamics rely on the administration of a bolus of contrast agent (CA) and the subsequent monitoring of the temporal distribution of the contrast agent in the tissue with an appropriate imaging modality and are often referred to as “bolus tracking” measurements. In addition to these bolus-tracking techniques, CEUS offers the unique method of “flash-replenishment” measurements. Here, imaging takes place not during the injection of CA, but afterwards, when a nearly constant concentration of CA is achieved. Dynamic information is obtained by disrupting the micro bubbles in the imaging plane with a pulse with high mechanical index, thus creating a “negative” bolus. Subsequently, micro bubbles are carried into the imaging plane by blood flow in the tissue, thus allowing the derivation of various hemodynamic tissue parameters from the dynamics of the replenishment.

The purpose of this study was to transfer the promising in vitro triplet therapy into an in vivo model using immunohistochemistry for evaluation of the response as gold standard. Additionally, we evaluated the in vivo response by detecting the anti-angiogenic and invasion-inhibiting effects of the serine protease inhibitor Upamostat®, the MMP inhibitor Ilomastat® and the selective COX-2 inhibitor Celecoxib® using the relatively new “flash replenishment” (FR) method for the assessment of tissue hemodynamics coupled with contrast-enhanced ultrasound (CEUS) in an experimental small-animal squamous cell carcinoma model.

## Methods

### Animal model and experimental protocol

The study was performed with the approval of the Institutional Committee for Animal Research in accordance with the guidelines of the National Institute of Health for the care and use of laboratory animals. All animal experiments were approved by the Bavarian state government (Application number: 55.2-1-54-2531-162-10).

*6 × 10*^*6*^*human hypopharynx carcinoma cells* were injected subcutaneously into the left abdominal flank of 22 female athymic nude rats (*Charles River*®, Sulzfeld, Germany/7-8 weeks old/180-220 g body weight). The animals were inspected daily to assess general appearance and tumor growth. When tumors reached a size of *~1.2 cm* in the largest probe based on caliper measurements in two dimensions (median seven days of subcutaneous tumor growth/SD two days), CEUS measurements were performed using a high-end ultrasound system (Siemens Sequoia 512®/ Acuson, Mountain View, Germany). For examinations, animals were anesthetized with intraperitoneal injections of *Ketamine*® (100 mg/kg bodyweight, *Ketavet*®*,* Pfizer Inc. *©*, New York, NY) and *Xylazine*® (10 mg/kg bodyweight, *Rompun*® 2%, Bayer, Leverkusen, Germany). A *22-gauge* butterfly catheter (B. Braun AG®, Melsungen, Germany) was inserted into a tail vein for the manual injection of contrast media. After tumor tissue was contrasted homogeneously, all microbubbles in the imaging plane were eliminated with a high-energy pulse. Subsequent replenishment of the microbubbles in the sonic plane was observed and recorded. To prevent motion artifacts, the transducer was not held by hand but was fixed in a dedicated device. The transducer position of the baseline scan was recorded photographically to allow reproduction of the same conditions for the follow-up measurements.

Both the treatment group and the control group received a daily application of solvents for a period of one week with the control group receiving solvents only while the solvents of the treatment group also included the triple therapy of Upamostat®, Celecoxib® and Ilomastat®. After six days of treatment, follow-up measurements were performed the next day using the identical measurement protocol to assess the effect of the triple therapy. Video sequences were exported and analyzed with VueBox® (Bracco Suisse®, Geneve, Switzerland) as described previously [20]. In addition, immunohistochemistry analyses (Proliferation/Ki-67, vascularization/CD31, apoptosis/caspase3) were performed to validate the CEUS measurements.

### Contrast-enhanced ultrasound (CEUS)

Technical developments over the past decade have focused on different microbubble consistencies as well as effective methods of detecting their non-linear signals. The low mechanical index allows the production of real-time gray-scale images. Contrast-specific techniques use a low applied acoustic pressure to produce images based on nonlinear acoustic interaction between the ultrasound system and stabilized microbubbles. These microbubbles oscillate and resonate, giving continuous contrast enhancement to gray-scale images. SonoVue® (Bracco®, Milan, Italy) is a second-generation contrast agent consisting of stabilized microbubbles of sulfur hexafluoride gas, allowing for direct and easy removal via the respiratory system. While low in solubility, it is innocuous, isotonic with human plasma and devoid of antigenic potential since it contains no proteinaceous component. The required dosage for a single injection was 0.3 ml followed by 0.3 ml of saline to improve the detection of contrast enhancement in the tumor tissue.

### Triple therapy

Upamostat® (Wilex®, Munich, Germany) at a concentration of 0.03 mg/kg in 0.1 ml stock (9.6 ml Aqua dest and 0.4 ml Ethanol), Ilomastat® (50 mg/kg in 0.1 ml Ethanol, US Biological, Massachusetts, U.S.A), and Celecoxib® (25 mg/kg in 0.1 ml Ethanol, Pfizer, Berlin, Germany) were administered to the animals via gavage each day. The control group received the same quantity of solvents without any drugs. In both groups, 0.2 ml Ulcogant (Merck®, Darmstadt, Germany) was then administered following the solvent treatment to block acid in the stomach.

### Data analysis with VueBox

Consensus reading data evaluation was performed in a blinded manner by an experienced radiologist (five years in-depth experience) and a physicist (main focus perfusion quantification, 6 years experience) using the digitally stored video sequence data sets for the analysis of the contrast-enhanced ultrasound examinations. The regions of interests (ROIs) were always drawn to the entire tumor and to a hypervascular tumor site because the tumor changed throughout treatment. Follow-up investigation was performed promptly to ensure that hypervascular areas were compared in identical tumor localization. To derive perfusion-related parameters from the flash-replenishment measurement, a lognormal model [24-27] was fitted to the time-intensity curves of the previously defined regions, yielding estimates for the relative blood volume (rBV_FR_), wash-in rate (WiR_FR_), mean transit time (mTT_FR_) and relative blood flow (rBF_FR_) as the ratio of rBV_FR_ and mTT_FR_.

### Immunohistochemistry

Consecutive cryosections (4 μm) of each tumor were fixed in acetone (10 min, RT) and incubated in H_2_O_2_ (10 min, RT, 0.03%) to block endogenous peroxidase activity. Subsequently, slides were incubated with either mouse anti-rat CD31 (1:100,2 h, Becton Dickinson®, Heidelberg, Germany), rabbit anti-human Caspase-3 (1:50; 2 h, Cell Signaling, Boston, MA, USA), mouse anti-human EpCAM VU1D9 (1:2000; 1 h, Cell Signaling, Boston, MA, USA) or mouse anti-human Ki67 (1:800;1 h, Dako®, Hamburg, Germany). Thereafter, sequential incubations with biotinylated anti-mouse, rat-adsorbed (for CD31, EpCAM and Ki67), anti-rabbit secondary antibody (for Caspase 3), and peroxidase-labelled avidin-biotin-peroxidase complex were conducted (Vector Lab. Inc.®, Burlingame, CA, USA). Amino-ethyl-carbazole (AEC) peroxidase substrate was used for the detection of antigen/antibody complexes indicated by red-brown staining. Counter-staining was achieved with hematoxylin (blue). Negative controls were conducted simultaneously using respective mouse/ rabbit isotype-control antibody (Cell Signaling). Finally, sections were mounted in Kaiser’s glycerol gelatin for subsequent analysis.

### Evaluation of immunostaining

Proliferation and apoptosis rate were measured as the percentage of Ki67 and caspase-3 positive cells amongst all tumor cells, respectively. Furthermore, vascularization was measured as the amount of CD31-positive vessels in the tumor. The results given are relative to control-treated rats, which we have evaluated in comparable groups under the same circumstances.

### Laser scanning fluorescence microscopy

The presence of Ki67-positive tumor cells and CD31-positive vessels in rat stroma tissue was analyzed using a fluorescence laser scanning system (TCS-SP2 scanning system and DM IRB inverted microscope, Leica®, Solms, Germany). Ki67 and CD31 staining were performed with specific antibodies as described above. Dye-coupled Alexa antibodies (Alexa-488 for Ki67 in green and Alexa-647 for CD31 in red; Molecular Probes, Eugene, USA) were used as secondary antibodies. Subsequently, Hoechst 33342 was used for labeling of nuclear DNA (Sigma®, Taufkirchen, Germany). Leica Confocal Software Lite (Leica®, Solms, Germany) was used for evaluation according to the manufacturer’s instructions.

### Statistical analysis

Continuous variables are presented as the mean and standard deviation or absolute and relative frequencies if appropriate. Given the parametric distribution, a paired Student’s T-test was employed to compare measurements obtained at baseline and follow-up, an unpaired Studient’s T-test was use for comparisons of continuous parameters between the therapy and control group. Also, median percentage changes between baseline and follow-up of CEUS parameters as well as for the immunohistochemical data of the control and treatment groups were calculated. Analyses were carried out using SPSS (IBM SPSS Statistics, USA, Version 20). P values of < 0.05 were considered to indicate statistical significance.

## Results

CEUS measurements were successfully performed without any technical difficulties in all 22 rats. Two animals died of anesthetic complications before the follow-up measurements. The mean examination time for the CEUS measurements was 6.9 minutes, excluding animal preparation. After seven days of median subcutaneous tumor growth (SD two days), the mean tumor volume based on two-dimensional caliper measurements was *~130 mm*^*2*^. No side effects due to administration of the contrast medium were observed.

### Tumor diameter: (therapy vs. control)

In the placebo group, the tumors grew aggressively with an average growth in cross-section from 1.36 cm^2^ to 2.74 cm^2^ on the last day of “treatment”. Tumor size increased by 101.4% on average. In the group that received the triple therapy, tumor size increased by only 52.8% on average with the average tumor 1.25 cm^2^ in cross-section at the beginning of the therapy growing to 1.91 cm^2^ at the end. The therapy resulted in a reduction of the increase in tumor size of 48.6%. This represents a statistically significant and strong reduction of tumor growth upon application of triple medication (p ≤ 0.05).

**Figure 1:**
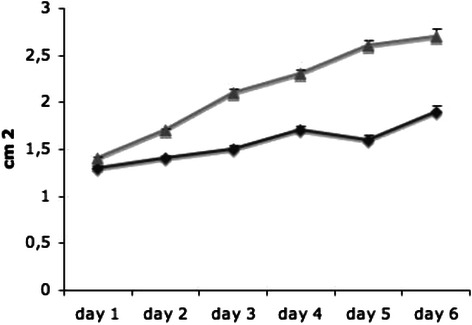
In the placebo group, the tumors grew aggressively in average crosssection from 1.36 cm^2^ to 2.74 cm^2^ on the last day of treatment (grey graph). The tumors size increased 101.4%. In the group that received the triple therapy, the tumor increase in size by 52.8%. The average tumor was 1.25 cm^2^ at the beginning of the therapy and grew up to 1.91 cm^2^ at the end (black graph). The therapy resulted in a reduction of the increase in tumor size of 48.6% (p ≤ 0.05).

In addition, 14 days after tumor implantation and after six days of therapy, the average tumor mass was 1.5 g in the treatment group and 2.1 g in the placebo group (p = 0.3) (see Figure 1).

### CEUS quantification using Vuebox

**Figure 2:**
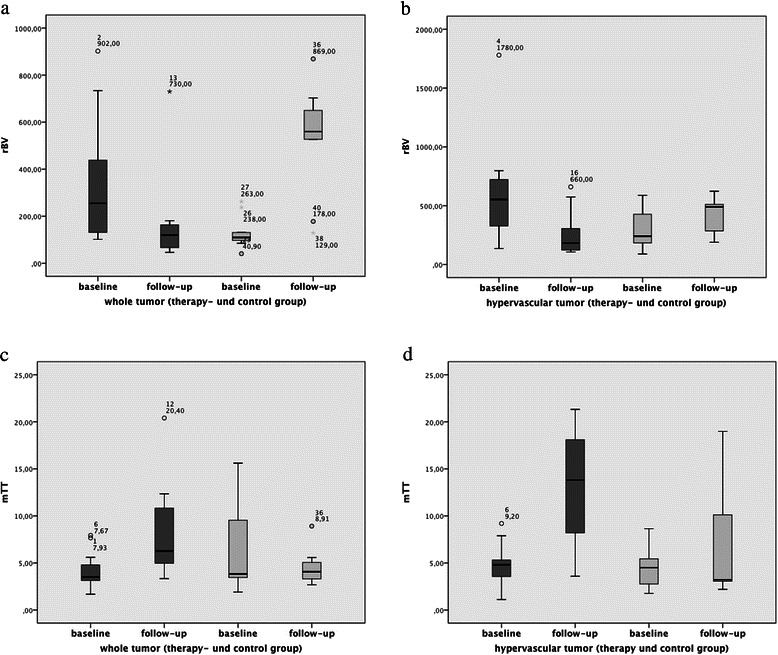
Therapy group : dark gray/Controll group : light gray. Subsequently, in the therapy group we observed a significant (p ≤ 0.01) decrease of the relative Blood Volume (rBV) between the baseline and follow-up measurements of the whole tumor (wt) **(a)** and of the hypervascular tumor (ht) areas **(b)** (p ≤ 0.01). The mean Transit Time (mTT) of the wt **(c)** and the ht **(d)** areas showed significant increase (p ≤ 0.003/0.001) in the follow-up measurements. In the control group, we observed a significant (p ≤ 0.01) increase of the rBV between the baseline and follow-up measurements of the wt and a minor increase (p ≤ 0.16) of the ht areas. The mTT of the wt and the ht areas showed no significant changes in the follow-up measurements.

**Figure 3:**
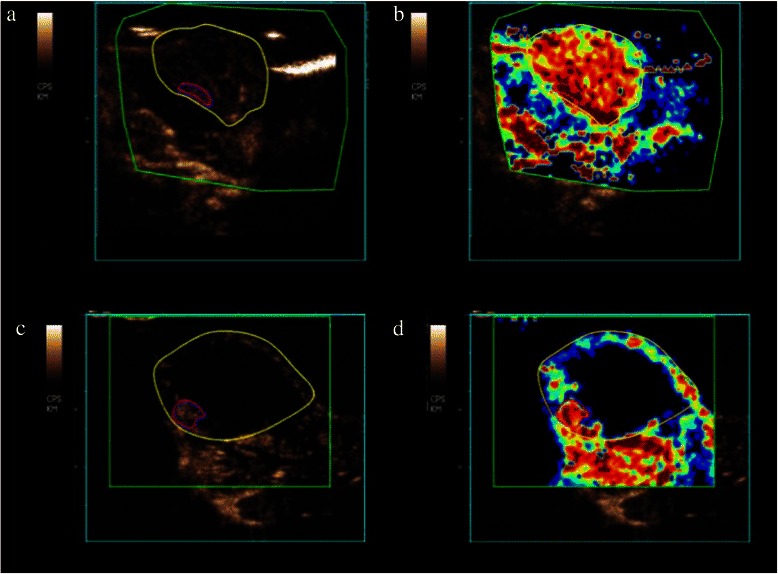
The contrast-enhanced ultrasound image shows a significant reduction of the vascularization between the baseline **(a)** and follow-up **(c)** scan. Although the tumor grew in size during treatment, must of the tumor is necrotic. The blood volume parameter maps show a corresponding decrease of the relative Blood Volume (rBV) within the remaining hypervascular tumor betwenn the baseline **(b)** and follow-up **(d)** scan.

In the control group, we observed a significant (p ≤ 0.01) increase of the relative blood volume (rBV) between the baseline and follow-up measurements of the whole tumor from 131.6 (SD 72.1) to 529.9 (SD 237.2) and a non-significant increase (p ≤ 0.16) of the hypervascular tumor areas from 320 (SD 183.5) to 414.9 (SD 51.1). In contrast, we observed a significant (p ≤ 0.01) decrease of the rBV between the baseline and follow-up measurements of the whole tumor from 338.3 (SD 272.8) to 164.3 (SD 193.7) and of the hypervascular tumor areas from 595.4 (SD 448.8) to 190.3 (SD 57.4) (p ≤ 0.01) in the therapy group. Please see also Figures 2 and 3.

The mean Transit Time (mTT) of the whole tumor and the hypervascular tumor areas showed no significant changes (p ≤ 0.182/0.338) in the follow-up measurements (baseline: 6.8; SD 5.2/follow-up: 4.5; SD 1.9//baseline: 4.7; SD 2.6/follow-up: 6.6; SD 6.6) in the control group. In contrast, in the therapy group the mTT of the whole tumor and the hypervascular tumor areas showed a significant increase (p ≤ 0.01/0.01) in the follow-up measurements (baseline: 4.2; SD 2/follow-up: 8.3; SD 5.1//baseline: 4.8; SD 2.3/follow-up: 13.2; SD 6.5). Please see also Figures 2 and 3.

In the control group a significant increase (p ≤ 0.01) of the Perfusion Index (rBV/mTT) was observed for the whole tumor (baseline: 30.2; SD 24.7/follow-up: 123.9; SD 63.7), while the hypervascular tumor areas (baseline: 91.2; SD 57.4/follow-up: 121.8; SD 95.2) revealed only a minor increase (p ≤ 0.376). A significant decrease (p ≤ 0.01/0.01) of the Perfusion Index was observed for the whole tumor (baseline: 102.6; SD 96.1/follow-up: 27.4; SD 39.3) and the hypervascular tumor areas (baseline: 177.2; SD 197/follow-up: 32; SD 50.9) in the therapy group.

The Wash-in Rate (WiR) displayed a minor increase of the whole tumor (p ≤ 0.596) and a significant (p ≤ 0.01) increase of the small hypervascular tumor areas (baseline: 21.6; SD 16/follow-up: 27.9; SD 25.8//baseline: 63.6; SD 39.8/follow-up: 154.3; SD 84.4) in the control group. In contrast, the WiR also displayed a significant decrease of the whole tumor (p ≤ 0.01) and the small hypervascular tumor areas (baseline: 37.5; SD 32.6/follow-up: 11.7; SD 8.3//baseline: 102.8; SD 76.8/follow-up: 25.5; SD 14.3) in the therapy group.

No significant differences were observed within each group (p > 0.05) or between the baseline measurements of the control and therapy groups. Please see also Table 1.

**Table 1:** **Absolute values of the whole tumor and the hypervascular area at baseline and follow-up for the therapy and control group (parameters: relative Blood Volume, mean Transit Time (s), Perfusion Index (rBV/mTT), Wash-in Rate)**

	Whole tumor					Hypervascular area				
	Baseline		Follow-up		P	Baseline		Follow-up		p
	Mean	SD	Mean	SD		Mean	SD	Mean	SD	
Therapy: relative Blood Volume	337.3	273	164.3	193.7	0.01	595.4	448.8	190.3	57.4	0.01
Control: relative Blood Volume	131.6	72	529.9	237.2	0.01	320.8	183.5	414.9	51.1	0.16
Therapy: mean Transit Time (s)	4.2	2.0	8.3	5.1	0.01	4.8	2.3	13.2	6.5	0.01
Control: mean Transit Time (s)	6.8	5.2	4.5	1.9	0.18	4.7	2.6	6.6	5.6	0.34
Therapy: Perfusion Index (rBV/mTT)	102.6	96	27.4	39.3	0.01	177.2	197.0	32.0	50.9	0.01
Control: Perfusion Index (rBV/mTT)	30.2	25	123.9	63.7	0.01	91.2	57.4	121.8	95.2	0.38
Therapy: Wash-in Rate	37.5	33	11.7	8.3	0.01	102.8	76.8	25.5	14.3	0.01
Control: Wash-in Rate	21.6	16	27.9	25.8	0.6	63.6	39.8	154.3	84.4	0.01

### Immunohistochemistry

Proliferation of the tumors treated with the triple therapy was 26% less in comparison to the placebo group (p ≤ 0.05). The apoptosis rate was 1.8 times higher in the therapy group compared to the placebo group (p ≤ 0.01).

**Figure 4:**
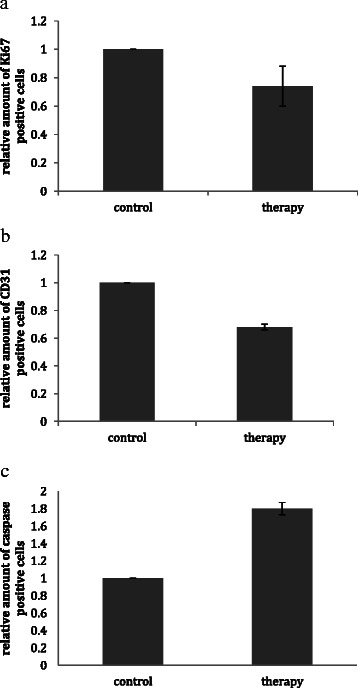
Immunohistochemistry revealed a significant decrease of the proliferation **(a)** and number of vessels **(b)** in the tumor and a significant increase of the apoptosis rate **(c)** in the treatment group compared with the placebo group.

**Figure 5:**
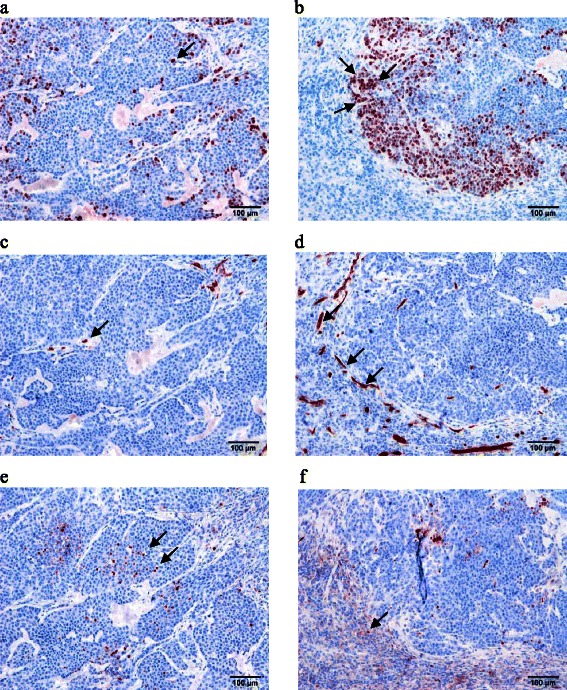
Immunohistochemistry revealed a significant decrease of the proliferation (red) in the therapy group **(a)** in comparison to the placebo group **(b)**. The number of vessels (red) in the therapy group **(c)** decreased in comparison to the untreated group **(d)** and we observed a significant increase of the apoptosis rate (red) in the treatment group **(e)** compared with the placebo group **(f)**.

**Figure 6:**
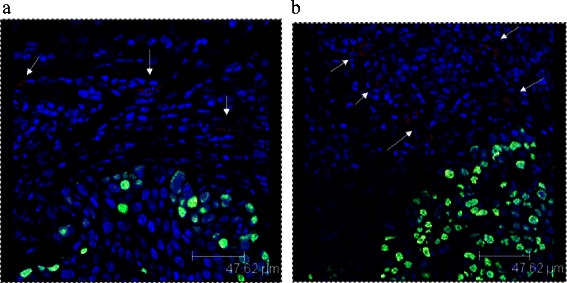
Figure 6 shows the expression of Ki67-positive (green) tumor cells and CD31-positive (red) vessels in the tissue analyzed using a fluorescence laser scanning system. Immunohistochemistry revealed a significant decrease of the proliferation and the number of vessels in the therapy group **(a)** in comparison to the placebo group **(b)**.

The number of vessels in the tumor was reduced by 32% in the triple therapy group compared with the placebo group (p ≤ 0.05) (see Figures 4, 5 and 6).

In particular, the proliferation rate was determined as the percentage of Ki67 positive cells among all cells in the tumor. Likewise, the apoptosis rate was measured as the percentage of Caspase3-positive cells. Furthermore, the vascularization was measured as the number of CD31 positive vessels in the appropriate tissue. All results are given relative to control-treated rats.

## Discussion

Previous reports disclosed that multimodal therapy strategies are more suitable to efficiently address the plethora of mechanisms underlying tumor progression than single therapeutics. Thus, the combination of inhibitors that target different aspects of tumor progression and metastasis formation is a promising concept [16,17].

We have previously demonstrated in vitro the efficacy of a combination of the serine protease inhibitor WX-UK1, the MMP inhibitor Ilomastat® and the selective COX-2 inhibitor Celecoxib® [18]. Triple medication reduced tumor cell invasion by 80% and neoangiogenic sprouting of HUVECs by 40%. In the present study, the potential of triple medication was assessed in a small-animal model of cancer. Head and neck carcinoma cells were xenotransplanted into athymic nude rats and tumor size, proliferation, and perfusion were analysed using contrast-enhanced ultrasound and immunohistochemistry. In the therapy group the proliferation was reduced by 26% (p ≤ 0.05). The apoptosis rate of the tumors treated with the triple therapy showed a significant increase of 1.8 times in comparison to the control group (p ≤ 0.01). As expected due to the pharmacological mechanism of action, the number of vessels in the tumor showed a significant reduction by 32% in the triple therapy group compared with the placebo group (p ≤ 0.05). The therapy resulted in an increase of apoptose and a decrease of prolifertation and angiogenesisa, indicating a reduction in tumor invasion, which was measured as a reduction in tumor growth. Thus, these results demonstrate the efficacy of triple medication in vivo and support the further transfer of knowledge to pre-clinical and clinical settings. Young et al. showed in their study with the cell line YD-10B (squamous cancer of the tongue) a dose-dependent inhibition of tumor growth and proliferation up to 60% with Celecoxib® alone [24]. An in vivo study using Celecoxib® in a mouse model in 2006 also demonstrated an increase of caspase three and nine and an increase of apoptoserate in the tumor cells, which resulted in a decrease in tumor growth [25].

Hawinkels et al. were also able to show a reduction of neoangiogenesis using the MMP inhibitor GM6001 and Marimastat using colorectal tumor cells in vitro [26]. A more direct and detailed comparison of our results to other studies was not feasible, because, to the best of our knowledge, there are no other studies that also include the described triplet therapy in an experimental small-animal squamous cell carcinoma model.

Since a histological follow-up of an anti-angiogenic therapy is clinically infeasible, as it is not appropriate for the patients to undergo repetitive biopsies within short time spans, we choose the CEUS method to assess effects on tissue hemodynamics as a surrogate for anti-angiogenic and invasion-inhibiting effects of the triple combination therapy.

We decided to use CEUS imaging because it is one of the most promising tools for imaging tumor angiogenesis and monitoring therapeutic effects of anti-vascular tumor therapy due to its lack of ionizing radiation, non-invasiveness, wide clinical availability and cost-effectiveness. The bolus tracking technique is a well-known and established technique that can be used in a wide range of modalities including CEUS, CT and MRI. However, due to the well-known disadvantages of this technique, e.g. the need to acquire an arterial input function in a blood vessel, we chose the flash replenishment method for the assessment of the tissue hemodynamics for our study. The flash replenishment method is specifically tailored to CEUS, and the results are independent of the injection speed or cycle time. Additionally, Paprottka and Ingrisch could demonstrate that although the lack of absolute, quantitative parameters hinders a direct comparison of both modalities, FR and BT are both suitable for relative comparison, e.g. between baseline and follow-up examinations [20].

Previous preclinical studies have shown that grey-scale ultrasound measurements of micro bubble contrast agent flow can be used to investigate tumor angiogenesis [19-23] to estimate the effects of antiangiogenic tumor therapy [19,27-36]. However, a direct comparison of our results to other studies was not possible because, to the best of our knowledge, there are no other studies that also include the described triplet therapy in an experimental small-animal squamous cell carcinoma model. As confirmed by the immunohistochemical procedure, the number of vessels in the tumor showed a significant reduction by 32% in the triple therapy group compared with the placebo group (p ≤ 0.05). Correspondingly, CEUS indicated a significant (p ≤ 0.01) decrease of the relative blood volume (rBV) and a significant increase of the mean transit time (mTT) in the therapy group between the baseline and follow-up measurements in both the whole tumor and the hypervascular tumor areas. Oppositely, the control group showed a significant (p ≤ 0.01) increase of the rBV in the whole tumor and a minor increase (p ≤ 0.16) of the rBV in the hypervascular tumor areas and no significant changes (p ≤ 0.182/0.338) of the mTT in either the whole tumor or the hypervascular tumor areas.

Our results indicate that quantitative detection of the tumor response during antiangiogenic treatment should be possible in the near future even in small tumors, i.e. tumors with a size of *~130 mm*^*2*^ or greater. Although the well-known limitations of ultrasound such as obesity, meteorism and noncompliance are also present for CEUS, due to the superficial position of the tumor and the intraperitoneal anesthetized injection, these limitations were of no consequence in our study. The study is, however, limited in direct applicability by the fact that our measurements were made in an experimental animal model under optimized experimental conditions such that the results may not be 100% transferable to clinical practice.

## Conclusion

The triple therapy is feasible and leads to a significant reduction by 32% of the number of vessels in the tumor in the triple therapy group compared with the placebo group, as proven by immunohistochemistry and a reduction of tumor growth of 48.6%. The anti-angiogenic and invasion-inhibiting effects of a triple combination therapy can be assessed non-invasively with CEUS using the “flash replenishment” (FR) method.
